# The Timing of Evolutionary Transitions Suggests Intelligent Life is Rare

**DOI:** 10.1089/ast.2019.2149

**Published:** 2021-03-10

**Authors:** Andrew E. Snyder-Beattie, Anders Sandberg, K. Eric Drexler, Michael B. Bonsall

**Affiliations:** ^1^ Mathematical Ecology Research Group, University of Oxford, Oxford, United Kingdom.; ^2^ Future of Humanity Institute, University of Oxford, Oxford, United Kingdom.

**Keywords:** Evolutionary transitions, Observation selection effects, Bayesian analysis.

## Abstract

It is unknown how abundant extraterrestrial life is, or whether such life might be complex or intelligent. On Earth, the emergence of complex intelligent life required a preceding series of evolutionary transitions such as abiogenesis, eukaryogenesis, and the evolution of sexual reproduction, multicellularity, and intelligence itself. Some of these transitions could have been extraordinarily improbable, even in conducive environments. The emergence of intelligent life late in Earth's lifetime is thought to be evidence for a handful of rare evolutionary transitions, but the timing of other evolutionary transitions in the fossil record is yet to be analyzed in a similar framework. Using a simplified Bayesian model that combines uninformative priors and the timing of evolutionary transitions, we demonstrate that expected evolutionary transition times likely exceed the lifetime of Earth, perhaps by many orders of magnitude. Our results corroborate the original argument suggested by Brandon Carter that intelligent life in the Universe is exceptionally rare, assuming that intelligent life elsewhere requires analogous evolutionary transitions. Arriving at the opposite conclusion would require exceptionally conservative priors, evidence for much earlier transitions, multiple instances of transitions, or an alternative model that can explain why evolutionary transitions took hundreds of millions of years without appealing to rare chance events. Although the model is simple, it provides an initial basis for evaluating how varying biological assumptions and fossil record data impact the probability of evolving intelligent life, and also provides a number of testable predictions, such as that some biological paradoxes will remain unresolved and that planets orbiting M dwarf stars are uninhabitable.

## 1. Introduction

Life on Earth has undergone a number of major evolutionary transitions (Smith and Szathmary, 1997). These include abiogenesis, as well as the emergence of increasingly complex forms of life such as eukaryotic, multicellular, and intelligent life. Some transitions seem to have occurred only once in Earth's history, suggesting a hypothesis reminiscent of Gould's remark that if the “tape of life” were to be rerun, “the chance becomes vanishingly small that anything like human intelligence” would occur (Gould, 1990). Here, we explore this hypothesis.

Given that we cannot rerun the “tape of life,” it is difficult to derive the probability of these major evolutionary transitions. An alternative would be to examine the timing and frequency of the transitions. The fact that eukaryotic life took over a billion years to emerge from prokaryotic precursors suggests it is a far less probable event than the development of multicellular life, which is thought to have originated independently over 40 times (Grosberg and Strathmann, 2007). The early emergence of abiogenesis is one example that is frequently cited as evidence that simple life must be fairly common throughout the Universe (Lineweaver and Davis, 2002). By using the timing of evolutionary transitions to estimate the rates of transition (probability per unit of time), we can derive information about the likelihood of a given transition even if it occurred only once in Earth's history.

However, an additional methodological challenge arises in estimating these rates from their timing in our evolutionary history, given that the timings are subject to a sample bias. In particular, we can only observe evolutionary transitions that occurred rapidly enough to fit within Earth's habitable lifetime. It is estimated that the increased luminosity of the Sun will make complex eukaryotic life impossible on Earth in about 0.8 to 1.3 billion years (Ga) (Caldeira and Kasting, 1992; Franck *et al.*, 2006). If a long period of time is required for intelligence to evolve, any intelligent observers on an Earth-like planet may observe early evolutionary transitions occurring regardless of the true transition rate.

A previous analysis that accounted for these sample biases found that early abiogenesis was still consistent with life being rare, as long as one did not presuppose a particular order of magnitude for the transition rate (Spiegel and Turner, 2012). Here, we generalize this Bayesian analysis to a chain of multiple evolutionary transitions, drawing inspiration from the work of Carter (1983) and others (Hanson, 1998; Carter, 2008; Watson, 2008; McCabe and Lucas, 2010) that sought to explain why intelligent life emerged so late in Earth's history. The transitions we consider include abiogenesis, eukaryogenesis, the emergence of sexual reproduction, and the emergence of language and intelligence, although the model can be applied more broadly. These particular transitions are selected because large scientific uncertainty remains around how frequent such transitions are, and because they were prerequisites for the existence of intelligent life on Earth.

## 2. Context

The issue of how likely life and intelligence are to emerge on planets in the Universe has long been a mainstay of the SETI-related debates, both as terms in the Drake equation (Vakoch and Dowd, 2015) and whether the endeavor is even rational (Ćirković, 2013).

While some of the attempts at bounding or estimating life are based on astrophysical considerations of planet formation, habitable zones, and other abiotic properties (Kasting *et al.*, 1993; Ward and Brownlee, 1999; Lineweaver *et al.*, 2004; Spiegel *et al.*, 2008; Lammer, *et al.*, 2009; Johnson and Li, 2012; Rushby *et al.*, 2013; Loeb *et al.*, 2016), some of the most contested probabilities deal with abiogenesis, and the emergence of complex life and intelligence. Estimates of the probability of abiogenesis per planet in the literature range from truly microscopic (due to the need to search combinatorially vast spaces) (Hart, 1980), over the small (values giving on the order of one civilization per observable Universe) (Blum, 1965), to modest (Lineweaver *et al.*, 2002), to so large that they predict life in nearly any habitable environment (De Duve, 1995; Halley, 2012). Similarly, the fraction of life-bearing planets with complex life (and intelligence) can be estimated to be very high (Sagan, 1963; Wallenhorst, 1981), moderate (1%) (Billingham *et al.*, 1979; Bounama *et al.*, 2007), or very low (Behroozi and Peeples, 2015). Indeed, for both one can find estimates in the literature and based on models spanning 100 orders of magnitude (Scharf and Cronin, 2016; Sandberg *et al.*, 2018).

One of the oldest arguments against SETI is the biological contingency argument (Simpson, 1964): the evolution of anything similar to humans has a minuscule probability since biological evolution is dominated by contingency, is radically open-ended, and has no determinism or tendency toward intelligence. Even in similar environments, the chance of getting “humanoids” is minimal, and most environments will be vastly different. This is the same argument used by Mayr in his debate with Sagan: out of the approximately 50 billion species on Earth, only humans evolved intelligence, suggesting a low probability (Mayr, 1995a, 1995b, 1995c).

Sagan (1995) countered by noting that if there are enough possible pathways, even individually very unlikely paths can in sum give a high probability of an intelligent outcome. He also noted that extrapolating from our case is either valid, and we should expect Earth to be an average sample, or it is improper to extrapolate, in which case Mayr's argument fails. While the biological contingency argument can be attacked in other ways, for example, by emphasizing convergent evolution (Puccetti, 1968; Morris, 2003), and supported by noting the *lack* of convergent evolution toward human-like intelligence in the fossil record (Lineweaver, 2009), the key issue is how representative the Earth's biosphere history is (Rospars, 2013).

### 2.1. The Carter argument

Intelligent life emerged on a timescale similar to that of Earth's lifetime. It took 4 Ga for intelligent life to emerge, and in perhaps less than 1 Ga, the increasing luminosity of the Sun will likely destroy Earth's ability to support complex life, due to increased surface temperatures (Franck *et al.*, 2006) and an eventual breakdown in the carbon cycle (Lenton and Bloh, 2001). Intelligent life therefore emerged on a timescale within an order of magnitude of our star's lifetime. This is puzzling, as the timescales associated with biological and stellar evolution are driven by fundamentally different processes and thus ought to be uncorrelated.

Carter (1983) noticed this coincidence and proposed a resolution to the puzzle based on observation selection effects. Letting τ_⊙_ denote the lifetime associated with our star, and τ${}_{\ell }$ be the timescale it takes for evolution to produce intelligent life, one can analyze three possibilities: τ_⊙_ ≫ τ${}_{\ell }$, τ_⊙_ ≈ τ${}_{\ell }$, or τ_⊙_ ≪ τ${}_{\ell }$, denoting the situations in which the lifetime of the star either greatly exceeds the timescale associated with intelligent life, approximately equals it, or is greatly exceeded by it. Carter argues that *a priori*, the possibility that τ_⊙_ ≈ τ${}_{\ell }$ is exceptionally unlikely, leaving τ_⊙_ ≫ τ${}_{\ell }$ and τ_⊙_ ≪ τ${}_{\ell }$ as realistic alternatives. We can also rule out τ_⊙_ ≫ τ${}_{\ell }$ with high probability, given that intelligent life did not emerge exceptionally early when compared with the Sun's lifetime. This brings us to the possibility that τ_⊙_ ≪ τ${}_{\ell }$. This would mean that most stars will never support intelligent life, as the star will burn out before such life emerges. However, in the rare locations in which intelligent life does emerge, it will find itself emerging within the lifetime of the star, and moreover is most likely to observe τ_⊙_ ≈ τ${}_{\ell }$, consistent with our own observations. Observation selection effects therefore explain why we see these timescales tightly coupled, even if such an outcome is *a priori* unlikely.

In the same article, Carter proposed a simple model of evolutionary transitions to describe the process of intelligent life emerging. The model proposes that intelligent life requires *n* “critical steps,” each of which occurs at some rate λ. He further stipulates that λ^−1^ > τ_⊙_, so that the probability per unit time of the critical step is low enough that the time it takes for each critical step will typically exceed the lifetime of the star. A number of interesting properties follow from this model. First, the probability that the final transition occurs at time *t* is proportional to *t^n^*, so that the final critical step is likely to occur toward the end of habitable time remaining. Second, the amount of time remaining will be roughly equal to τ_⊙_/(*n* + 1), allowing one to estimate the number of critical steps that occurred in Earth's evolutionary history simply by knowing the amount of time left in Earth's habitable lifetime.

When Carter originally proposed the model, it was thought that the biosphere could last for another 4 Ga, which in turn suggested that there were likely only one or two critical steps in our evolutionary history. Subsequent improvements in climate models led to additional research that suggested that the time remaining is substantially shorter, on the order of 1 Ga (Caldeira and Kasting, 1992). A number of researchers have returned to Carter's critical step model and re-estimated the number of critical steps predicted by the remaining lifetime of the biosphere. Watson (2008) found that the best fit was with four critical steps, while Carter (2008) suggested between five and six. Waltham (2017) went further to demonstrate that models up to 12 critical steps still fall within a 95% confidence interval. Using the Carter model without further hard steps [*e.g.*, just abiogenesis, as in Lineweaver *et al*. (2002) and Spiegel and Turner (2012)] produces significantly different estimates from including hard steps (Flambaum, 2003). The hard step model can also be combined with estimates of the window length (Lingam and Loeb, 2019), or even possible early windows for abiogenesis that later close (Lineweaver and Davis, 2003).

Here, we quantify the Carter argument in Bayesian terms. Rather than hold λ fixed and estimate *n* as done in past literature (*i.e.*, estimate the number of critical steps while assuming λ^−1^ ≫ τ_⊙_), we hold *n* fixed and estimate λ (*i.e.*, determine what the timing of each evolutionary transition says about its rate). This has the advantage of quantifying the data, priors, and/or assumptions that would be needed to overturn the Carter argument. Quantifying the Carter argument also helps highlight exactly how strong the argument holds. Frank and Sullivan (2016) argue that as long as the odds that intelligent life emerges on a habitable planet are >1 in 10^24^, we will not be alone in the observable Universe. However, we find that for reasonable priors, the Carter argument places substantial probability on the odds being <1 in 10^24^.

## 3. A Simplified Model of Evolutionary Transitions

### 3.1. The generalized Carter model

Let us assume that intelligent life requires a sequence of *n* evolutionary transitions. We assume that the transitions must occur sequentially, so that the second transition cannot occur until after the first one, the third not until after the second, and so on. Let *x_i_* be the timing of the *i*th transition, and let *t_i_* be the time it takes between the *i*th transition and the previous one, so that *t_i_* = *x_i_* − *x_i_* _− 1_. We set *x*_0_ = 0, representing the earliest possible time that the first transition could have occurred. We assume that once an evolutionary transition is possible (*i.e.*, once the previous transition has occurred), it occurs at a constant average rate *λ_i_*, so that each *t_i_* is exponentially distributed with an expected transition time of *β_i_* = 1*/λ_i_*.

The joint probability density function for the transition times is the product of exponentials:


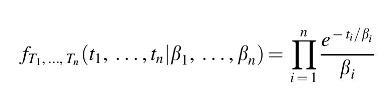


We can calculate the probability that all of the transitions successfully occur before the lifetime of Earth using the cumulative distribution function of the final transition time *F_X_n__*(*x_n_*|*β*_1_,…,*β_n_*). This is done by using the properties of the hypoexponential distribution (Amari and Misra, 1997), which we describe in the Appendix.

### 3.2. A Bayesian analysis of transition times

Our objective is to estimate evolutionary transition rates, given how long it took to complete each transition. This can be found by using a Bayesian update as follows:


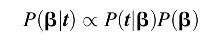


where *t* is the sequence of transition times *t*_1_,…,*t_n_*, *β* denotes our *β* parameters, and *P*(**β**) is a prior density over the expected transition time parameters. The term *P*(*t*|**β**), the probability of observing transition times *t* given the parameters **β**, is equivalent to the likelihood function as follows:

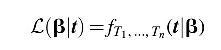



However, this likelihood function needs to be renormalized to account for the fact that we can only observe these data if all evolutionary transitions occurred before the end of Earth's lifetime. Accounting for this sample bias can be done by dividing the likelihood *L*(**β**|t) by the probability that all transitions occurred within the lifetime of Earth. If *L* is the lifetime of Earth, then our adjusted likelihood function is as follows:


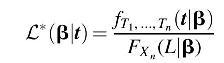


where 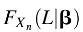 is the probability that all transitions occur before the end of Earth's lifetime.

### 3.3. Limiting behavior of the likelihood

We can use limits to evaluate the likelihood of expected transition times that are arbitrarily large. Setting all rate parameters equal, so that all $\beta_{i}=\beta_{j}$, one can show that for a model with *n* transitions, taking the limit as *β* → ∞ results in a likelihood of the following:


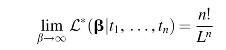


A more detailed proof of this limit is provided in the Appendix. This nonzero constant highlights our first key result, which is that the likelihood does not diminish to zero even as the expected transition times go to infinity. Extremely improbable evolutionary transitions will still be observed by intelligent life if such transitions are crucial to the existence of such intelligence, and this holds true no matter how improbable the transition event.

## 4. The Data and Priors

The model requires data for the starting and ending points for Earth's habitability, as well as the timing of the four transitions we evaluate (the origin of life, eukaryotes, sex, and intelligence). Earth's oceans are thought to have formed between 4.4 and 4.2 billion years ago (Gya) (Peck *et al.*, 2001; Cavosie *et al.*, 2005), which we take to be the earliest point at which life could form on Earth. Our earliest evidence of life dates between 4.1 and 3.5 Gya (Bell *et al.*, 2015; Awramik, 1992), while the earliest evidence of eukaryotic life has been dated between 1.6 Gya and almost 1.9 Gya (Parfrey *et al.*, 2011; Betts *et al.*, 2018). Sexual reproduction has been hypothesized to have emerged concurrently with eukaryotic life, with firm evidence of its existence at 1.2 Gya (Butterfield, 2000). We date the arrival of human language, symbolic reasoning, and intelligence to be approximately present day, with the arrival of *Homo sapiens* at 200,000 years ago and the earliest artwork around 80,000 years ago (Pääbo, 2014). We assume the habitability of Earth will end in 0.8–1.3 Ga due to the increasing luminosity of the Sun. We summarize these data in Table 1.

**Table 1.** Data for Evolutionary Transition Timing and Biosphere Start/End Dates

Transition	Date, Gya	Source	Method
Abiogenesis	>4.10 ± 0.01	Bell *et al.* (2015)	Carbon isotope ratio
Abiogenesis	3.9	Betts *et al.* (2018)	Molecular clock
Abiogenesis	>3.86 ± 0.01	Mojzsis *et al.* (1996)	Carbon isotope ratio
Abiogenesis	>3.77–4.28	Dodd *et al.* (2017)	Microfossils, isotope ratio
Abiogenesis	>3.5	Awramik (1992)	Microfossils, stromatolites
Cyanobacteria	<10 Ma	Lazcano and Miller (1994)	Molecular evolution model
Eukaryotes	<1.84	Betts *et al.* (2018)	Molecular clock
Eukaryotes	>1.87–1.68	Parfrey *et al.* (2011)	Molecular clock
Sex	>1.2	Butterfield (2000)	Fossils of red algae
Intelligence	≈0	Pääbo (2014)	Oldest artwork

Boundary	Timing	Source	Method
Oceans form	4.40 Gya	Peck *et al.* (2001)	Oxygen isotope ratio
Oceans form	4.3–4.2 Gya	Cavosie *et al.* (2005)	Oxygen isotope ratio
Biosphere ends	0.8–1.2 Ga	Franck *et al.* (2006)	Climate model
Biosphere ends	0.9–1.5 Ga	Caldeira and Kasting (1992)	Climate model
Biosphere ends	≈1.0 Ga	Kasting (1988)	Climate model
Biosphere ends	1.8–3.3 Ga	Rushby *et al.* (2013)	Climate model

Biosphere end dates are given for eukaryotic life. Extremophiles may persist beyond the dates given.

### 4.1. The priors for transition rates

The prior distributions express subjective beliefs and uncertainties about evolutionary transition times, which are then updated to a posterior distribution based on the observed data. Given the large scientific uncertainties surrounding the “true” rate for each transition (abiogenesis, eukaryogenesis, sexual reproduction, evolution of intelligence), we begin by considering an uninformative prior: a log-uniform distribution for each transition rate. Following Spiegel and Turner (2012), this is equivalent to saying we have no prior information that informs us of even an order-of-magnitude estimate of each transition time (*β_i_*). In addition to being uninformative, the log-uniform prior is thought to be most appropriate given that it is also invariant to the choice of parameterization of event frequencies such as the mean waiting time for an event (*β*) or the mean number of events per unit time (*β*^−1^). In contrast, a uniform prior on mean waiting time *per se* would, for an interval bounded by 10^−10^ and 10^10^ Ga, imply ≈0.9 confidence in values near the upper bound, *>*10^8^ Ga, while a uniform prior over the same interval expressed in frequency terms would imply ≈0.9 confidence in values near the lower bound on mean waiting times, *<*10^−8^ Ga.

To be well defined, each log-uniform distribution needs upper and lower bounds. Note that the selection of these bounds introduces an assumption that is no longer “noninformative.” To create an exceptionally conservative lower bound, we assume each expected evolutionary transition time cannot be faster than a rapid bacterial doubling time (roughly 10^−14^ Ga). Appropriately conservative upper bounds on expected transition times are more difficult to produce without controversy. Although some evolutionary transitions could be the result of incremental and deterministic processes, they could also require a precise combination of extremely rare events.

### 4.2. Combinatorial models for upper bounds

In general, if an evolutionary transition requires a specific combination of *N* binary elements, transition rates to any particular state decline as 2^−*N*^. Protein folding is one example that can serve as a more general analogy for why extremely long transition times should be considered. Folding of a 300 residue sequence can be naively modeled as a random search through a space of over 10^285^ conformational states (the bond between a given pair of residue is described by *φ* and *ψ* torsional angles, each typically regarded as occupying one of three low-energy conformations). Given this, it would take $\gg 10^{200}$ times the present age of the universe for a particular folding to occur, even assuming a sampling rate of 1 trillion conformational states per molecule per second and a volume of concentrated protein solution the size of Earth's oceans. As protein sequences have evolved to fold reliably, most proteins typically fold within seconds, driven by a so-called funnel in the free-energy landscape (Dill and Chan, 1997). However, a prebiotic world would have no evolutionary process to shape such a funnel (*i.e.*, variation with selection), and perhaps no reliable mechanism to assemble polymeric components of the requisite kinds. If so, then expected transition times for abiogenesis could be truly immense.

The transition to eukaryotic life also involves similar “chicken and egg” difficulties, with uncertainty on how an archaeon acquired a proto-mitochondrion, since endocytosis requires complex machinery only present in eukaryotes (Lane, 2011). A second potential hurdle for eukaryogenesis was the survival of the first prokaryotic host with a bacterial symbiont. Without the protection of spliceosomes and a nucleus, the prokaryotic host would be disrupted by extensive intron transfer from the lysis of its symbionts, resulting in few functional proteins (Koonin, 2011). The chimera cell would need to evolve these complex defenses faster than the mutation ratchet effect driving the (already tiny) population to extinction, which could have also required a rare specific outcome among a vast combinatorial space.

Combinatorial models could apply to the evolution of language and intelligence as well. Human language is thought to be fundamentally different than other forms of animal communication, and fundamental to our general intelligence via the human usage of the merge operation (the ability to combine two items into an unordered set) and the resulting ability to construct hierarchal and recursive expressions (Berwick and Chomsky, 2016). If hierarchal and recursive language only results after obtaining a specific combination of neutral alleles, the probability could be extremely low that each allele would spread to fixation and combine to result in intelligence. Some argue that language acquisition was subject to selection pressure, similar to the gradual evolution of the eye (Pinker and Bloom, 1990), but others argue that language arose suddenly (Bolhuis *et al.*, 2014), and that its origin was a consequence of biological spandrels or exaptations (Tattersall, 2008). Even more pessimistic models might incorporate fitness costs associated with large brains, both metabolically and in terms of high levels of parental care (Mayr, 1994; Lineweaver, 2009).

In the presence of this wide uncertainty, we proceed by initially setting the upper bound of each before 10^10^ Ga and calculating the posterior distribution. We subsequently examine what happens if we change the priors to be even more conservative and discuss whether such conservative priors are plausible.

## 5. Results of the Model, Data, and Priors

**FIG. 1.**
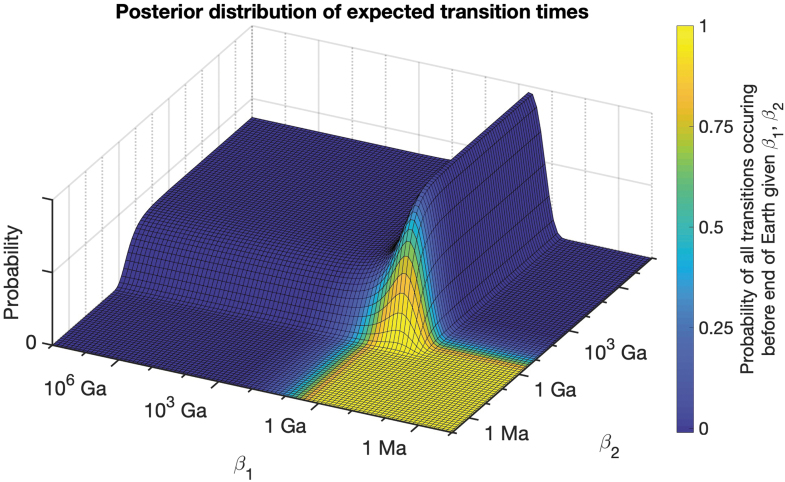
Posterior distribution of expected transition times in a two-step model (abiogenesis with expected time *β*_1_ and the emergence of human intelligence with expected time *β*_2_). Parameter combinations of rapid rates of transition, such as those resulting in expected transition times of 1 Ma, are inconsistent with the fossil record data and thus have a posterior probability close to zero. Conversely, expected transition times exceeding 10^6^ Ga are compatible with the data, with posterior probability asymptoting along a nonzero constant as the transition times approach infinity. As a result, parameter combinations resulting in intelligent life before the end of the Earth constitute a very narrow slice of posterior probability (marked in yellow). For example, <3% of posterior probability is assigned to parameters that result in the final transition occurring before the end of Earth's lifetime with a >1% chance (second row in Table 2, which also provides the data used for this figure). Color images are available online.

Our Bayesian calculation is repeated for a variety of combinations of evolutionary transitions (Fig. 1 and Table 2). The posterior probability of expected transition times is maximized around the transition time found in the fossil record (*e.g.*, if abiogenesis took 1 Ga, then posterior probability is maximized along values of *β*_1_ = 1 Ga). Dramatically fast rates are assigned very low posterior probability (*e.g.*, we can confidently rule out that an evolutionary transition that took 1 Ga does not have an expected transition time of 0.1 Ga). However, the calculation produces an interesting asymmetry, since dramatically slow rates are not assigned low probability in the same way. In fact, the fossil record data are consistent with expected transition times that exceed observed transition times, even by many orders of magnitude (with posterior probability leveling off at a nonzero value when all *β_i_* become sufficiently large). Expected transition times can become arbitrarily large while still being consistent with observed transition times, as suggested by the earlier results of nonzero likelihood in the limit when transition times went to infinity. This phenomenon is caused by observation selection effects, since even astronomically rare transitions will be observed if they were prerequisites for intelligent observers. Failing to account for this selection bias produces results that almost guarantee that all evolutionary transitions will occur within 100 Ga.

**Table 2.** Evolutionary Transition Times and Implied Posterior Probability of Reaching Final Transition

Transition times, Gya							Posterior weight $F\left(x_{n}\right)>\ldots$		
0th	1st	2nd	3rd	4th	L	N	10^−2^	10^−12^	10^−24^
4.3	4.2	—	—	0	5.8	2	0.068	0.40	0.83
4.2	3.8	—	—	0	5.2	2	0.026	0.25	0.68
4.2	3.8	1.8	—	—	5.2	2	0.041	0.27	0.70
4.2	—	1.8	—	0	5.2	2	0.012	0.18	0.61
4.3	4.2	1.8	—	0	5.8	3	0.0064	0.11	0.43
4.2	3.8	1.8	—	0	5.2	3	0.0022	0.059	0.30
4.2	3.8	1.8	1.2	—	5.2	3	0.0056	0.080	0.34
4.2	3.8	1.8	1.2	0	5.2	4	0.0003	0.014	0.11
4.3	4.2	1.8	1.7	0	5.8	4	0.0027	0.056	0.26
						2	0.42	0.66	0.94
						3	0.27	0.51	0.84
						4	0.18	0.39	0.71
							Prior weight $F\left(x_{n}\right)>\ldots$		

A table of evolutionary transition combinations and for each, a proportion of posterior probability assigned to parameters that achieve the final transition with probability >10^−^^2^, 10^−1^^2^, and 10^−^^2^^4^

As the number of included transitions increases, the prior and posterior probability of reaching the final transition falls.

The importance of this result is that the vast majority of the parameter range consistent with the fossil record is inconsistent with expecting the final transition to occur within the window of Earth's habitability, suggesting that intelligent life is highly improbable. For a model with two transitions, over 90% of the posterior probability is assigned to rate parameters that would result in less than a 1% chance of achieving the final transition within Earth's lifetime (Table 2). This proportion of parameter space increases to 99% in a three-step model and a four-step model (Table 2). A substantial amount of posterior probability is even assigned to combinations of transition rates that have a less than 10^−12^ or 10^−24^ chance of reaching the final transition within the time that Earth is habitable (corresponding to on the order of 10^−12^ stars in our galaxy or 10^−24^ stars in the observable universe). For example, a three-transition model has over 90% of posterior probability assigned to rates that have less than a 10^−12^ chance of reaching the final transition (Table 2). If complex or intelligent life beyond Earth requires analogous evolutionary transitions, then the fossil record combined with uninformative priors suggests that such life is exceptionally rare.

### 5.1. Priors required to change the result

Our posterior estimates favored the hypothesis that the selected evolutionary transitions were exceptionally rare. However, the strength of this result will change depending on the Bayesian prior. Increasing (or decreasing) the amount of prior probability assigned to long expected transition times (*e.g.*, by increasing or decreasing the upper bounds on the prior) will increase (or decrease, respectively) the amount of posterior probability assigned to long transition times. This is because the fossil record data are equally consistent with long transition times or extremely long transition times, and thus, only the prior rather than the data determines to what extent such extremely long transition times should be considered. Bayesian priors are meant to capture wide scientific uncertainty and *a priori* assumptions, so any upper bound, including our arbitrary selection of 10^10^ Ga, will be subject to controversy. It is perhaps more instructive to determine how conservative the priors would need to be to reach the conclusion that the evolutionary transitions should be expected within Earth's lifetime, and then ask whether limiting the prior in such a way would be reasonable.

We can calculate how low the upper bound of each prior would need to be to produce 10% or 50% of posterior weight on parameter values that predict intelligent life within Earth's lifetime with a greater than 1% or 10% chance (Table 3). To get such a result, the bounds need to be unrealistically small (*e.g.*, setting a maximum transition time of 40 Ga for a three-step model to get 10% weight with a greater than 10% chance of life). Given the enormous uncertainties around the processes underpinning these evolutionary transitions, it seems excessively conservative to claim we should limit our priors in such a way.

**Table 3.** Upper Bound Needed on the Log Uniform Before Getting a High Probability of Intelligent Life

10% of posterior weight such that			50% of posterior weight such that	
*n*	*P*(life) >1%	*P*(life) >10%	*P*(life) >1%	*P*(life) >10%
2	10^4.8^ Ga	10^3.1^ Ga	10^1.8^ Ga	10^1.2^ Ga
3	10^2.4^ Ga	10^1.6^ Ga	10^1.2^ Ga	10^0.8^ Ga
4	10^1.8^ Ga	10^1.2^ Ga	10^1.0^ Ga	10^0.6^ Ga

Data used for transition dates were 4.2 Gya for start of habitability window, 3.8 Gya for abiogenesis (used in all three models), 1.8 Gya for eukaryotes (used when *n* = 3 and *n* = 4), 1.2 Gya for sexual reproduction (used when *n* = 4), and a total habitability window of 5.2 Ga. The other transition in all models was considered to be the evolution of intelligence close to present day.

### 5.2. Data required to change the result

Discovering a second independent instance of an evolutionary transition (*e.g.*, a branch of life unrelated to our universal common ancestor) would be a dramatic development that would change our estimates of transition rates. If we assume the transition rate remains constant over time, we can include such information in a Bayesian update, multiplying the previous posterior distribution by a likelihood function that incorporates the new data. This likelihood function is *L*(*β_i_*) = (1 − *e*^−1*/βi*^)*τ*, the probability that one or more additional transitions occurred in time *τ*, where *τ* is the time window in which the transitions could have occurred (specifically, after the prerequisite transition but before present day).

**FIG. 2.**
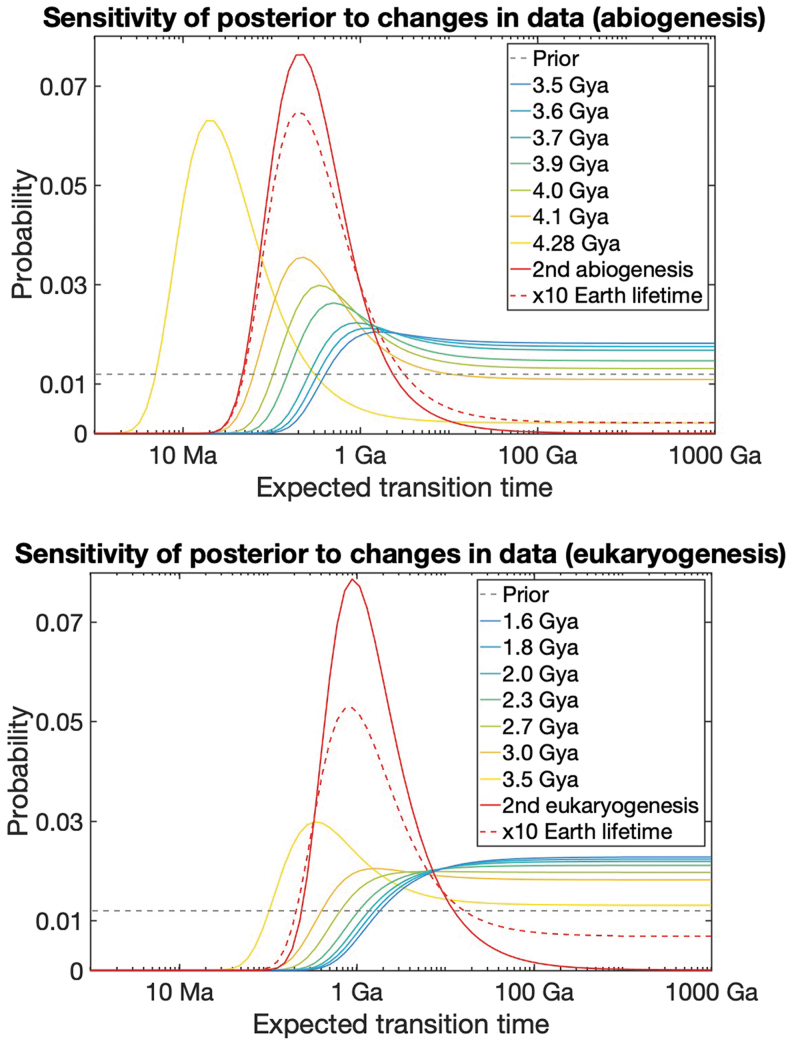
Posterior distributions of abiogenesis (top) and eukaryogenesis (bottom) transition rates for different timings of abiogenesis and eukaryogenesis. Earlier evidence of transitions pushes some posterior mass to faster transition rates but excluding exceptionally long expected transition times requires finding a second independent instance of the transition or the discovery that Earth will remain habitable for much longer than expected. If we were to find evidence of life occurring within 5 Myr of the start of Earth's habitability, this would also be enough to conclude that abiogenesis is not extremely rare. Color images are available online.

Notably, the likelihood of extremely large *β_i_* goes to zero with evidence of additional transitions (Fig. 2, red solid line). Discovering additional independent instances of a transition therefore rules out the possibility that a particular evolutionary transition is exceptionally unlikely. Indeed, multicellular life is thought to have emerged over 40 times, demonstrating that it is not an astronomically rare evolutionary transition. Finding even earlier evidence of a successful evolutionary transition could also change our estimates. We conduct a sensitivity analysis of our posterior estimates to changes in the fossil record data for abiogenesis and eukaryogenesis (Fig. 2). The primary effect of earlier evidence is to adjust the maximum likelihood peak (Fig. 2, yellow to blue spectrum). However, excluding the possibility of extremely long expected transition times requires an exceptionally rapid transition (on the order of 10s of millions of years). For example, finding evidence of eukaryotic life 3 billion years ago would still be insufficient to rule out expected transition times exceeding 1000 Ga (Fig. 2). The reason for this is that the conclusion holds so long as the habitable lifetime of Earth is roughly within the same order of magnitude as the evolutionary transition time. If it turned out that Earth will naturally remain habitable far longer than current science predicts, this would also be sufficient to overturn the conclusion that any of the transitions are rare (Fig. 2, red dotted line).

Given the wide uncertainty in the fossil record for when certain evolutionary transitions occurred, we can also examine what happens if we update our priors based on an interval of possible transition times rather than a specific transition time. Let *a_i_* be the lower end of the range of possible transition times for the *i*th transition (*e.g.*, the fastest plausible transition time perhaps only tentatively supported by the fossil record), and *b_i_* be the upper end of the range (*e.g.*, a conservative estimate where fossil evidence is clear). To update on these intervals, we use the following likelihood function:


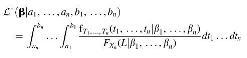


**FIG. 3.**
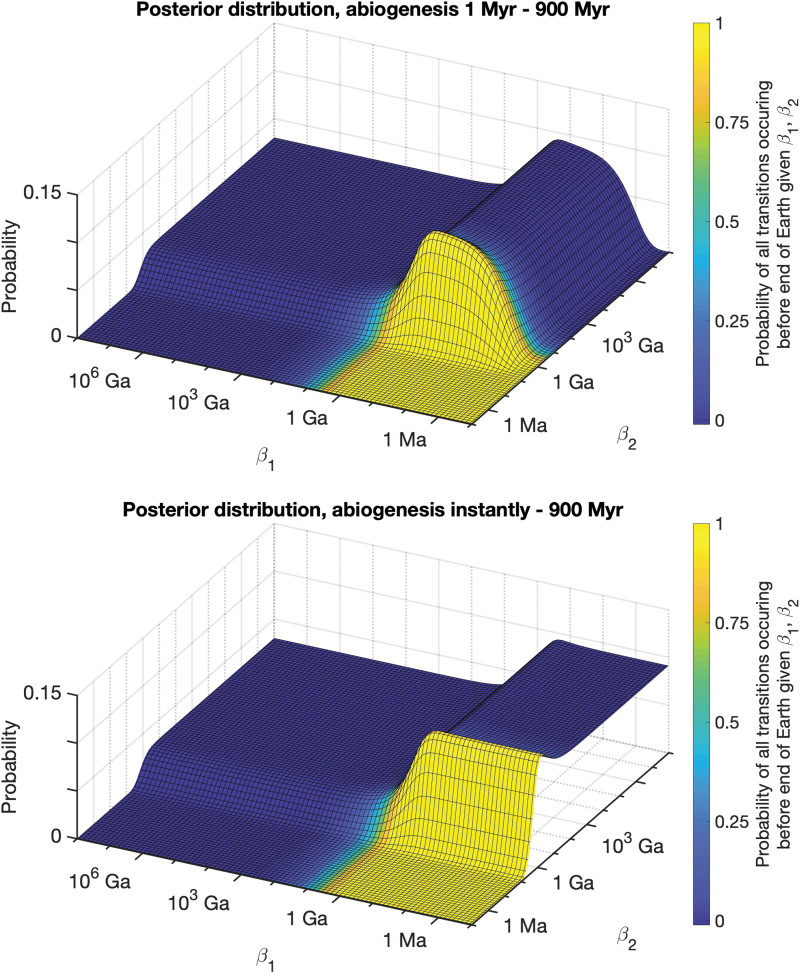
Posterior distributions updated on an interval of possible timings for the origin of life and eukaryotic life. On the top, we assume abiogenesis took between 1 and 900 Myr to occur, and on the bottom we assume that abiogenesis may have taken an amount of time ranging from instantaneously to 900 Myr. Although more posterior probability mass is assigned to rates that have a high probability of intelligent life emerging (denoted in yellow), the majority of posterior mass is still on rates that are incompatible with intelligent life occurring within the habitable lifetime of Earth (denoted in blue). Color images are available online.

In cases where the fossil record cannot definitively rule out an exceptionally short (or even instantaneous) transition time [*e.g.*, the origin of life or sexual reproduction, see Pearce *et al.* (2018)], this can cause the posterior estimates to include more weight on rapid transition rates (Fig. 3, top). When abiogenesis could take between 0 and 900 Myr, our posterior is consistent with abiogenesis being common or rare, replicating the results of Spiegel and Turner (2012). However, even when abiogenesis is common, our posterior still suggests that intelligent life is rare, supporting the Rare Earth Hypothesis (Ward and Brownlee, 1999). For a two-step model with abiogenesis taking between 0 and 900 Myr and eukaryotes taking between 800 and 2800 Myr, only 16% and 54% of posterior probability weight are assigned to parameters that would result in the transitions occurring successfully within the lifetime of Earth with >1% and 10^−12^ chance, respectively.

### 5.3. Assumptions required to change the result

Our model makes a number of simplifying assumptions, primarily that each evolutionary transition has a constant probability of happening per unit time throughout Earth's history. Although this is crude, we believe this is the best model because it requires the fewest biological assumptions. Still, it is worth discussing where the constant probability assumption is likely to fall short and to what extent our results rely on this assumption.

There are a number of reasons why the probability of an evolutionary transition could change over time. Perhaps most importantly, some evolutionary transitions may have required high oxygen concentrations as a source of energy, and oxygen concentrations have changed dramatically over Earth's history (Holland, 2006). The fact that oxygen concentrations have became high enough to support humans only in the past 800 Myr or so has led to some speculation that a planetary oxygenation time is the primary rate-limiting step to intelligent life (Catling *et al.*, 2005). Relatedly, complex life on land requires shielding from ultraviolet radiation, and the emergence of an ozone layer has also been hypothesized to be a rate-limiting step that is correlated with stellar evolution, undermining Carter's original argument (Livio, 1999).

**FIG. 4.**
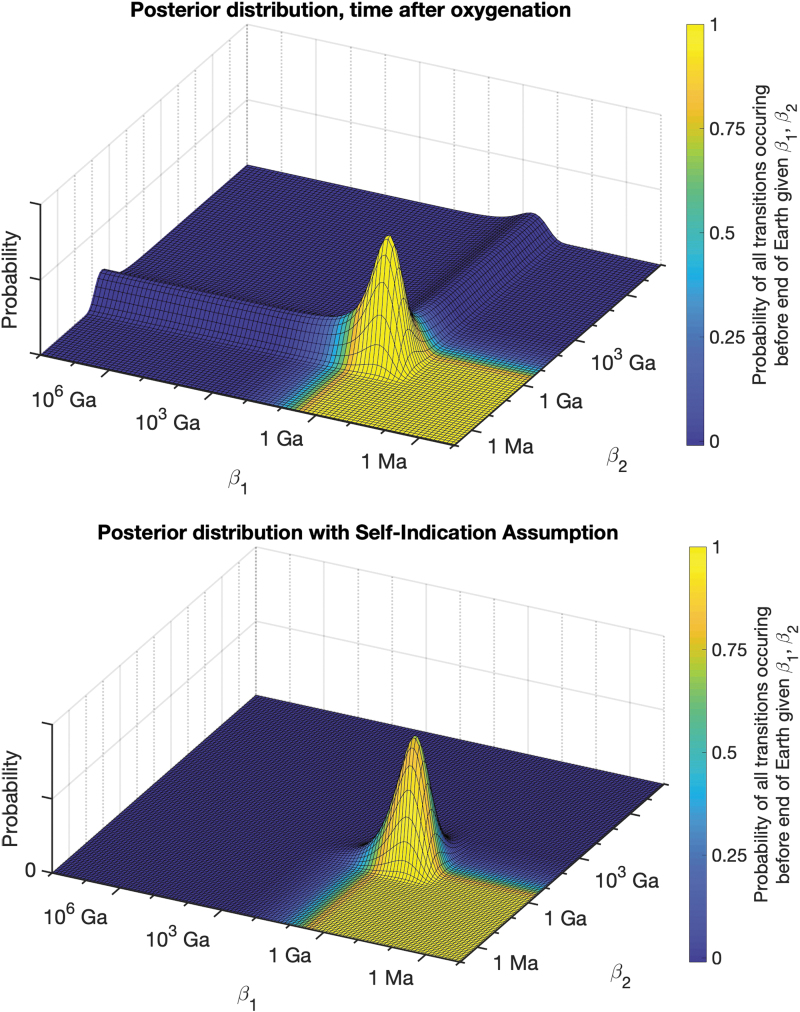
(Top) Posterior distribution if we assume two transitions that were made possible only after high oxygenation levels. Given the late oxygenation of Earth's atmosphere, these transition times are short, resulting in higher posterior probability on faster rates. However, arbitrarily slow rates are still not excluded. (Bottom) Posterior distribution if we adopt the self-indication assumption, and weight all parameter combinations by their probability of obtaining intelligent life. Only parameters that are consistent with intelligent life are assigned high probability, and extremely slow rates are ruled out entirely. Color images are available online.

To test this, we adjust our model so that the transition rates change over time. The most dramatic example of this is a model in which the final evolutionary transition to intelligent life has a probability of zero until vertebrates on land emerge (0.34 Gya), and that transition has probability zero until Phanerozoic oxygen concentrations are reached (0.8 Gya). This model essentially tells us that these transitions occurred fairly rapidly once oxygen concentrations were high enough, and the results show a much larger peak around fast rates, suggesting a higher probability of intelligent life emerging in the right conditions (Fig. 4, top). However, even these faster transition times are not enough to exclude extremely slow rates. For example, using the previous log-uniform prior with a lower bound of 10^10^ Ga still results in over 60% of the posterior parameter weight on rates that result in intelligent life with probability less than a 1 in 10^12^. Overall, accounting for a changing environment in terms of oxygen concentrations does not seem to be sufficient to overturn our key results.

There are numerous other ways in which the evolutionary transition rates could change over time. For example, the total biomass or the number of lineages relevant for a particular evolutionary transition could change over time (*e.g.*, the concentration of potential symbionts for eukaryotic life or the number of candidate animal lineages that could evolve intelligence), as could the rate of catastrophes that could act as evolutionary setbacks (*e.g.*, asteroid impacts during the Late Heavy Bombardment or the rate of gamma ray bursts) (Ćirković *et al.*, 2009). Although these examples make it clear that the assumption of a constant transition rate over time is oversimplified, creating a comprehensive model that incorporates all factors into the evolutionary transition rates would require many more assumptions. We can say in general that any alternative model still needs to explain why certain evolutionary transitions took such a long period of time.

To conclude that intelligent life is common, an alternative model would not only need to explain why intelligence emerged on roughly the same timescale of Earth's habitable lifetime, but also why eukaryotic life and other evolutionary transitions did so. Certainly with enough assumptions one could create a model that guarantees that each transition will occur at roughly the same time that it did on Earth, and then conclude that any Earth-like habitat lasting 5 Ga will have a high probability of hosting intelligent life. However, we ultimately think that the most parsimonious model is the one in which the long transition times are a byproduct of contingency.

In addition to assumptions about how evolutionary transitions occur, we also consider assumptions around how to use the information that we exist as observers. So far, we have assumed that we can derive no information on the probability of intelligent life from our own existence, since any intelligent observer will inevitably find themself in a location where intelligent life successfully emerged regardless of the probability. Another line of reasoning, known as the “Self-Indication Assumption” (SIA), suggests that if there are different possible worlds with differing numbers of observers, we should weigh those possibilities in proportion to the number of observers (Bostrom, 2013). For example, if we posit only two possible universes, one with 10 human-like civilizations and one with 10 billion, SIA implies that all else being equal we should be 1 billion times more likely to live in the universe with 10 billion civilizations. If SIA is correct, this could greatly undermine the premises argued here, and under our simple model it would produce high probability of fast rates that reliably lead to intelligent life (Fig. 4, bottom). However, embracing SIA leads to a number of other very counterintuitive results, such as essentially guaranteeing that the universe is exceptionally large or infinite even without accounting for cosmological evidence (Bostrom and Ćirković, 2003), or giving substantial probability to any bizarre theory that proposes a large enough population of observers to overwhelm the *a priori* implausibility of the theory (*e.g.*, a theory that each planet has 10^10^100 copies of itself on “other planes” would seem hard to refute if one adopted SIA) (Olum, 2002). Adopting SIA thus will undermine our results, but also undermine any other scientific result that would suggest a lower number of observers in the Universe. The plausibility and implications of SIA remain poorly understood and outside the scope of our present work. We proceed by proposing a set of testable predictions.

## 6. Testable Predictions

The model offers a number of testable predictions. First, we conclude that intelligent life is exceptionally rare and that we may possibly be the only intelligent civilization within the observable universe, so long as we assume that intelligent life elsewhere requires similar evolutionary transitions. Although this may seem like a large assumption, there are good reasons to believe that many evolutionary transitions have universal properties (Levin *et al.*, 2017). It also follows if we reason that our civilization is typical. If there were substantially easier evolutionary pathways to intelligent life that did not require such evolutionary transitions, we should expect to observe this easier evolutionary history instead. Although it is hard to show beyond doubt the absence of extraterrestrial intelligence, so far all of our astronomical data are consistent with being alone (Tipler, 1980). A handful of other testable predictions follow from the model as well.

### 6.1. Exceptionally rare transitions

The unlikeliness of different evolutionary transitions can also be tested more directly as we learn more about the underlying physical and biological processes of different evolutionary transitions or find evidence that a transition occurred more than once. Abiogenesis and eukaryogenesis both involve unexplained or partially unexplained paradoxes which could be resolved through further research. Examples include Eigen's paradox, describing the mystery of how systems for error correction evolved in the absence of error correction, with similar “chicken and egg” problems in the case of eukaryogenesis. Our model predicts that these paradoxes and similar ones may only be resolved by allowing for exceptionally rare chance events. If subsequent research discovers relatively easy pathways for each of these transitions (as it has done for multicellular life, for example), this would falsify our prediction (Chen and Kipping, 2018).

### 6.2. Habitability of red dwarf stars

There is an interesting connection between the evolutionary transitions model and arguments for the habitability of planets orbiting red dwarf stars (Rushby *et al.*, 2013; Waltham, 2017).

**FIG. 5.**
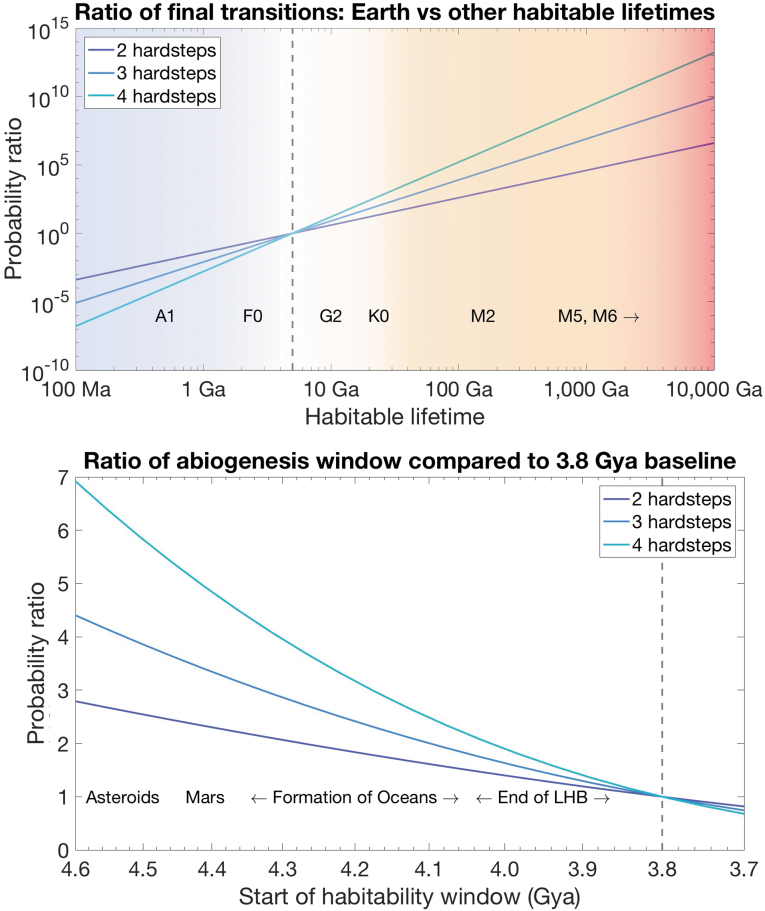
(Top) The ratio of final transitions that occur within a 5 Ga habitability window as opposed to a habitable window of another length of time, all else being equal. Habitable environments that last longer are more likely to support the final transition by many orders of magnitude, suggesting that other factors must reduce the habitability of red dwarf stars by many orders of magnitude. (Bottom) Probability ratios of earliest time abiogenesis could have occurred compared with a 3.8 Gya baseline. All else being equal, longer habitability windows have slightly higher probabilities, increasing the credence that the solar system was habitable from an early date (including an extraterrestrial origin of life). Color images are available online.

A typical M dwarf star will last 1 trillion years, roughly two orders of magnitude longer than our Sun, and could also be host to habitable exoplanets (Haqq-Misra *et al.*, 2017). However, the habitability of planets orbiting M dwarf stars remains an open question, as tidal locking, solar flares, or other harsh conditions could make these planets inimical to complex life (Zendejas *et al.*, 2010). If we start with the assumption that environments around dwarf stars are just as habitable as our solar system, such that the transition parameters are the same, we can calculate the probability ratio of final evolutionary transitions occurring around such dwarf stars as opposed to a habitat lasting as long as Earth. These probability ratios are heavily skewed in favor of longer lasting environments, by between 4 and 10 orders of magnitude depending on the number of transitions (Fig. 5, top). Assuming we are typical for intelligent life and finding ourselves not orbiting a red dwarf star, our model brings us to a testable prediction that the transition parameters are not similar, and some other factor is reducing the habitability of dwarf star environments by a factor of over 10,000 when compared with the Earth. The strength of this predicted factor is great enough that the prediction ought to be testable when using climate models or other tools currently accessible, and coincide with eventual scientific consensus that dwarf stars are inimical for complex life.

### 6.3. Extraterrestrial origin of life?

When was the earliest that life could have emerged? Given our model, we can evaluate this by taking the likelihood ratio between the hypothesis of an early conducive environment (say, with the formation of the oceans at 4.3 Gya) and the hypothesis that life was only possible relatively late in Earth's beginning (say, with the end of the Late Heavy Bombardment at 3.9 Gya). It has also been suggested that Mars was habitable 100 million years before Earth's oceans formed (Sleep and Zahnle, 1998), and that asteroids could have been habitable yet another 100 million years earlier (Abramov and Mojzsis, 2011). If the rate of material transfer between Mars and Earth is high enough to consider the two planets a single environment, we can compare the likelihood ratio between an extraterrestrial origin of life and origin of life on Earth as well. In general, the model favors hypotheses with earlier possible starting dates for the first transition (Fig. 5, bottom), with the strength increasing with the number of transitions included (Davies, 2003; McCabe and Lucas, 2010). The effect is very modest though. For example, the likelihood ratio for a 4.4 Gya starting point versus a 3.8 Gya starting point differs by only a factor of about two to seven. Perhaps more relevant is a prediction that if we were to find life on Mars, it would have emerged extremely early and have a common ancestor with life on Earth.

## 7. Conclusions

It took approximately 4.5 billion years for a series of evolutionary transitions resulting in intelligent life to unfold on Earth. In another billion years, the increasing luminosity of the Sun will make Earth uninhabitable for complex life. Intelligence therefore emerged late in Earth's lifetime. Together with the dispersed timing of key evolutionary transitions and plausible priors, one can conclude that the expected transition times likely exceed the lifetime of Earth, perhaps by many orders of magnitude. In turn, this suggests that intelligent life is likely to be exceptionally rare. Arriving at an alternative conclusion would require either exceptionally conservative priors, finding additional instances of evolutionary transitions, or adopting an alternative model that can explain why evolutionary transitions took so long on Earth without appealing to rare stochastic occurrences. The model provides a number of other testable predictions, including that M dwarf stars are uninhabitable, that many biological paradoxes will remain unsolved without allowing for extremely unlikely events, and that, counterintuitively, we might be slightly more likely to find simple life on Mars.

## Abbreviation Used

SIA: Self Indication Assumption
SETI: Search for extraterrestrial intelligence
LHB: Late Heavy Bombardment
Ma: million years
Ga: billion years
Mya: million years ago
Gya: billion years ago

## Appendix

### The Generalized Carter Model

Let us assume that intelligent life requires a sequence of *n* evolutionary transitions. We assume that the transitions must occur sequentially, so that the second transition cannot occur until after the first one, the third not until after the second, and so on. Let *x_i_* be the timing of the *i*th transition, and let *t_i_* be the time it takes between the *i*th transition and the previous one, so that *t_i_* = *x_i_* − *x_i_* _− 1_. We set *x*_0_ = 0, representing the earliest possible time that the first transition could have occurred. We assume that once an evolutionary transition is possible (*i.e.*, once the previous transition has occurred), it occurs at a constant average rate *λ_i_*, so that each *t_i_* is exponentially distributed with an expected transition time of *β_i_* = 1*/λ_i_*. The joint probability density function for the transition times is the product of exponentials:

The probability that *n* transitions successfully occur before a certain point in time (*e.g.*, the lifetime of Earth) can be found by computing the cumulative distribution function
